# Supplementary figures and images for: Genomic analysis of transcriptional networks directing progression of cell states during MGE development
Source: Neural Dev. 2018 Sep 14;13:21. doi: 10.1186/s13064-018-0119-4 (PMC6138899; doi:10.1186/s13064-018-0119-4)

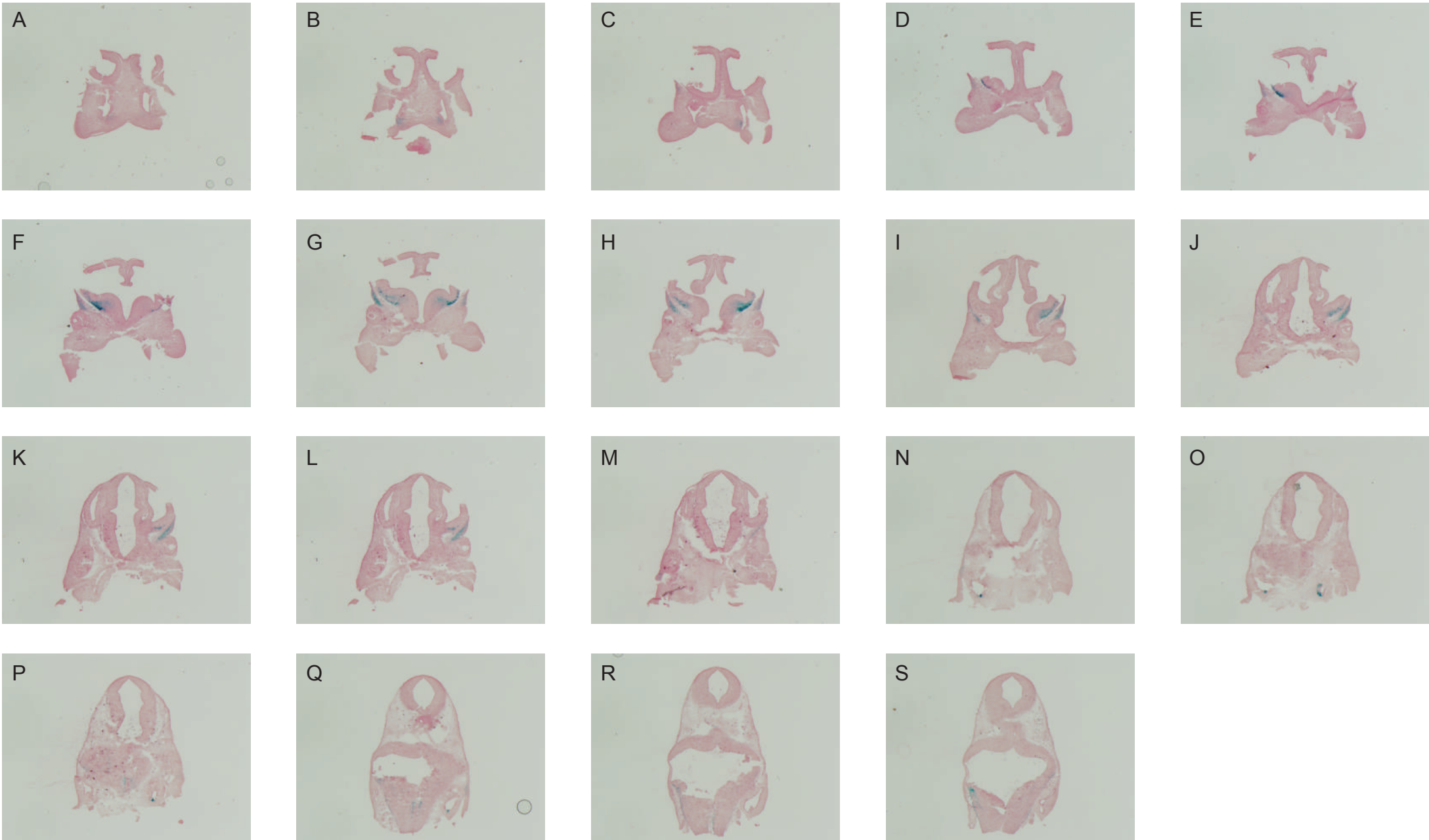

Supplement: Supplementary file 2 — Forebrain activity of hs623. Coronal sections of the hs623 transgene showing its activity in forebrain at E11.5. The sections are arranged rostral (A) to caudal (S). (PDF 135 kb) [file 13064_2018_119_MOESM2_ESM.pdf]

# *Tcf12* - e13.5

A

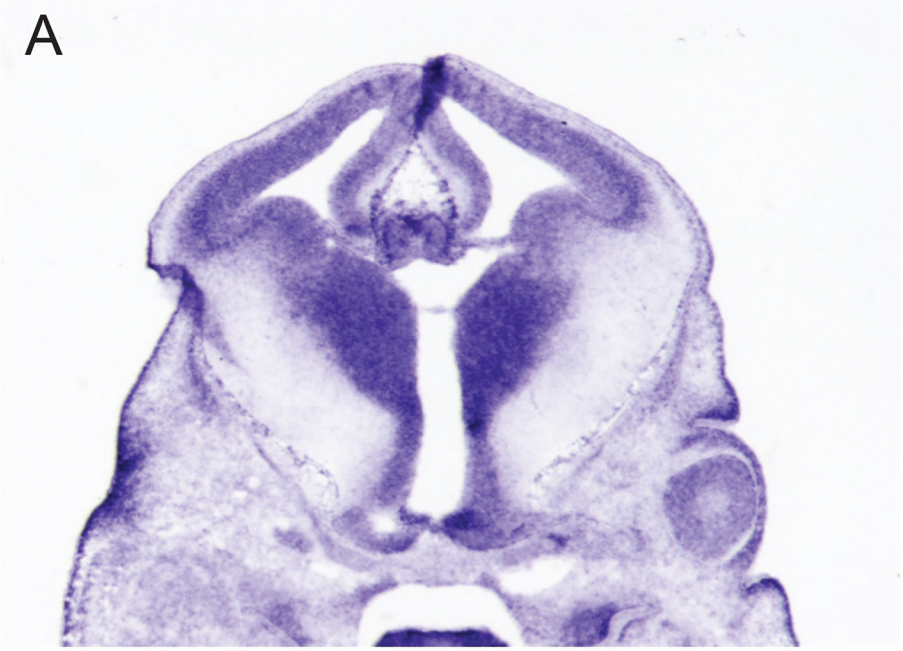

B

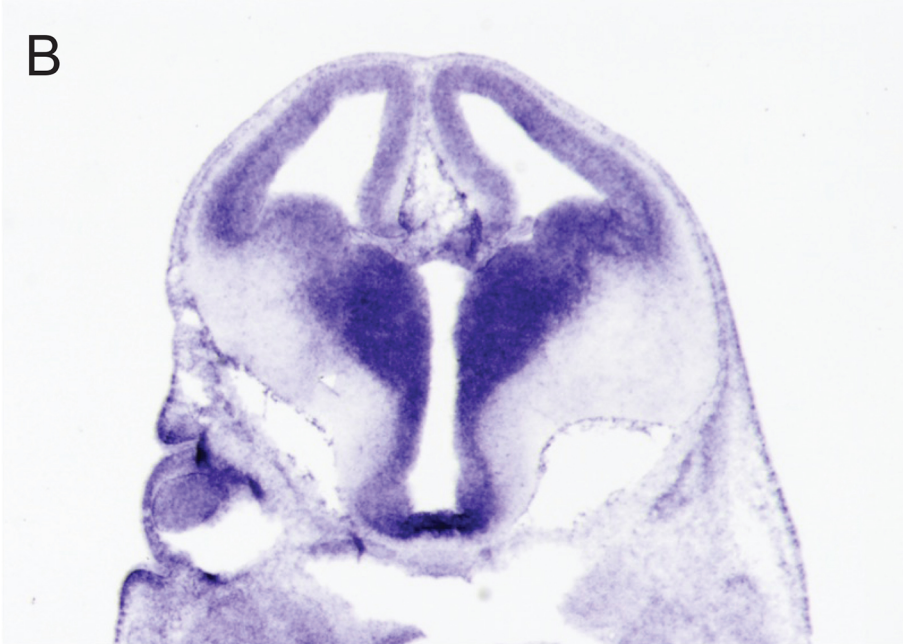

Supplement: Supplementary file 4 — Tcf12 expression in Sox6 conditional knockout. In situ analysis of Tcf12 in WT (A) and Sox6 conditional knockout (B) forebrain at E13.5. (PDF 4383 kb) [file 13064_2018_119_MOESM4_ESM.pdf]
