# Supplementary material for: Genomic analysis of transcriptional networks directing progression of cell states during MGE development
Source: Neural Dev. 2018 Sep 14;13:21. doi: 10.1186/s13064-018-0119-4 (PMC6138899; doi:10.1186/s13064-018-0119-4)
Supplement: Supplementary file 3 — DNA sequences of hs623KO founder mice. DNA sequence of the modified hs623 locus in the five founder mice carrying the hs623 deletion. (PDF 65 kb) [file 13064_2018_119_MOESM3_ESM.pdf]

Sequences of hs623KO founders.

Sequence 5’ of deletion.

Sequence 3’ of deletion.

Deleted sequence in founder.

Sequence hs623KO founder #1:

>MS_432601-502_1-1-TOPO-_T7_G01.ab1
NNNNCNNNNGCNGCTCGAGCGGCCGCCAGTGTGATGGATATCTGCAGAATTCGCCCTTGAGCAGTGTTATTTAAAAAAAT
CACCACACACACACACACACACCCTCAAACTGTGCATTTTATAATTAGGCTTTTGTCATTGTTGCTGTTGGCCTAAGCAA
GTGATTTTCAATTACAGTAAGCACACCAACCTCTCCAGAGTGATCTACTGGGGCAGACTTAAGAGGGAAAAAAATTAATG
ACAGCAATGAAACCATCTCTAGAAAAAAACCTGAAAGCCACCTAGAATTTAATTGTCAGGAAAACAAGCAGCAAAGGAGA
ATTGAATCTGTTTTAAGCTCACACAATTCAGAAATAAAGTGTATCTTAATCTAGTGTGAGGTGGAGACAAAATCTGTTAA
GAAAGAGGCATATAATATCCTAGAGAGAGGGGCAAGAACCTGATGAAGGGCGAATTCCAGCACACTGGCGGCCGTTACTA
GTGGATCCGAGCTCGGTACCAAGCTTGATGCATAGCTTGAGTATTCTATAGTGTCACCTAAATAGCTTGGCGTAATCATG
GTCATAGCTGTTTCCTGTGTGAAATTGTTATCCGCTCACAATTCCACACAACATACGAGCCGGAAGCATAAAGTGTAAAG
CCTGGGGTGCCTAATGAGTGAGCTAACTCACATTAATTGCGTTGCGCTCACTGCCCGCTTTCCAGTCGGGAAACCTGTCG
TGCCAGCTGCATTAATGAATCGGCCAACGCGCGGGGAGAGGCGGTTTGCGTATTGGGCGCTCTTCCGCTTCCTCGCTCAC
TGACTCGCTGCGCTCGGTCGTTCGGCTGCGGCGAGCGGTATCAGCTCACTCAAAGGCGGTAATACGGTTATCCACAGAAT
CAGGGGATAACGCNNGAAAGAACATGTGAGCAAAAGGCCAGCAAAAGGCCAGGAACCGTAAAAAGGCCGCGTTGCTGGCG
TTTTTCCATAGGCTCCGCCCCCCTGACGAGCATCACAAAAATCGACGCTCAAGTCAGANGTGGCGAAANCCGACAGGACT
ATAAAGATACCAGGCGTTTCCCCCNNNNGCTCCCTCNNGCGNNCNNCNGNTNCGACCCNNNNCTTANNNNNCNNNCNNNT
TNNCCNNTNNGGGANCGNGNGCTTTCTCNNANCTCANGCNNNANGNNNTCTCANNNGNNNNNNGNCNNNNNNNNNNNNGN
CNNNNNNNNCNNNNCCNCNNTNNCANNCCNNANCCGNNNNNNNNNCNNNNN

>mm9_dna range=chr9:71822294-71824207 5'pad=500 3'pad=500 strand=+
repeatMasking=none
TTATGTAAAGTAAATCCATCTGGATGTTTGCTCCATCCCATACTAGAAAC
CTGTGATCCCCAACCAGAATGAGTTATAGGAAAGCTGCAGGCAAAATTGT
CTCCATACAGCACAATCCATTATCCCTGTCAAGTTAATGTTCCGTGTTGA
GCCATATGGCACGGGTACACATTCAACAAAGGAAATTCCTTAAACTTGTT
ATAATCAGCACTTGCAGTCATCCAAAAAACTGACATAAACAGCTTTATTG
TGTTTAGCACATTTCAGTTAATATCCATGACAAAAACAGGAAACAGTACA
GGAAAAGGAGGAGGAATGTGGGGAGCAGTGTTATTTAAAAAAATCACCAC
ACACACACACACACACCCTCAAACTGTGCATTTTATAATTAGGCTTTTGT
CATTGTTGCTGTTGGCCTAAGCAAGTGATTTTCAATTACAGTAAGCACAC
CAACCTCTCCAGAGTGATCTACTGGGGCAGACTTAAGAGGGAAAAAAATT
AATGACAGCAAT**TTTCCATGGTATGAGCAAAACTAAACCTTGAAATTCTA**
GTGATTCAGATAAATCAGTTCTGGCAAAATAACAAAACTCTAAATCCTTA
TATTTGTTTTCTTTCTACAGTCATTTTATAATTGTTAAAATGCAACTGAG
CTTTATTAAGGAAAAAGAAACCAACGCAATTTAAGTAAAGTATCTTCATA
TGCATTTAAGAGCAGGTCACAGAAAATCAAGGGCTCCCACAAGCCTTTGG
TACTCGGCACATCAACTCTAGTCAAGGTATAAGTAGAGCAGAACTTCATT
TCAGCCCCCGCAGTGACAACAATAAAATGCTGAGAAAACACACTATCAAA
TACTGAACTCAGCATCAATTTCTGTTAATTACAGCCTTGTCAGCAACGTG
ATTATTCAAACAACTCATGCAAATGTTAATGAGGCCTTATTTGCATATTT
ATTTTTTCCTTTGTTGAAATGTCATTGATTATTACATTCTACTATGATGA
ATGTGGCTGATGATGTGCTCTGATATGTAATTAGTCATTAGGTAGAATGA
ATAGATCAATTATGAAAAAGGACTAAGATTAAGAAATATCAAACAAGACC
GCCCAACGTAACTATAGAATCTTTTTTTAAAAGGACCTAATAAATAATTT
CTTGACATTTAAACTGCAACATCAGTAGTTTTCGATAAATAAAGGACTAC
TTTTGTGAGGTTAAATGAAGGCTTAAAAATACATCAGTACTCTGATAATC
CAATTACGATCACAGAAACCATCTCTAGAAAAAAACCTGAAAGCCACCTA
GAATTTAATTGTCAGGAAAACAAGCAGCAAAGGAGAATTGAATCTGTTTT
AAGCTCACACAATTCAGAAATAAAGTGTATCTTAATCTAGTGTGAGGTGG
AGACAAAATCTGTTAAGAAAGAGGCATATAATATCCTAGAGAGAGGGGCA
AGAACCTGATGGAGAATTACTATTCATAAAACAGGGTTAGCCAAAAAAAA
AAAACCAAAAAAACAAAAAAACAAAAAAAAAAACCAAAACCCCTTTACATG

GTGACTTGTGCTCAGAGGAAAGGATTATTTAGCTCCTGAAACAGTTTGCT
GTAACTATCCAGCCAGTACTTGCTATTCGCAGTTGAAAAAAAAAAAAAAA
AAACCACATTACTGCAGACCAGATGGGAACATTCCACATGACCAAACCAA
TCAATCACCTCGGTGGGGCGCAGGTGCAACACAGCCGTGTAGCAACACAC
CATTTCACAGTCTATCGGGCACAAACACATGGCCACCAATCAATGGCACC
CTGAGGACCTCGGAGGGGGCAGGCTCTCTGGAGTCTTACTGGTCCATGCC
TGATGACTTCATAATCACACATCATTTCATTCACTAGAGGATACAAACTA
GCATTCTAATAACC

Sequence hs623KO founder #2:

>MS_432601-503_1-2454-topo-_T7_H01.ab1
NNNNNNNNNNGCATGCTCGAGCGGCCGCCAGTGTGATGGATATCTGCAGAATTCGCCCTTGAGCAGTGTTATTTAAAAAA
ATCACCACACACACACACACACACCCTCAAACTGTGCATTTTATAATTAGGCTTTTGTCATTGTTGCTGTTGGCCTAAGC
AAGTGATTTTCAATTACAGTAAGCACACCAACCTCTCCAGAGTGATCTACTGGGGCAGACTTAAGAGGGAAAAAAATTAA
TGACAGCAATTTTCCATCTCTAGAAAAAAACCTGAAAGCCACCTAGAATTTAATTGTCAGGAAAACAAGCAGCAAAGGAG
AATTGAATCTGTTTTAAGCTCACACAATTCAGAAATAAAGTGTATCTTAATCTAGTGTGAGGTGGAGACAAAATCTGTTA
AGAAAGAGGCATATAATATCCTAGAGAGAGGGGCAAGAACCTGATGAAGGGCGAATTCCAGCACACTGGCGGCCGTTACT
AGTGGATCCGAGCTCGGTACCAAGCTTGATGCATAGCTTGAGTATTCTATAGTGTCACCTAAATAGCTTGGCGTAATCAT
GGTCATAGCTGTTTCCTGTGTGAAATTGTTATCCGCTCACAATTCCACACAACATACGAGCCGGAAGCATAAAGTGTAAA
GCCTGGGGTGCCTAATGAGTGAGCTAACTCACATTAATTGCGTTGCGCTCACTGCCCGCTTTCCAGTCGGGAAACCTGTC
GTGCCAGCTGCATTAATGAATCGGCCAACGCGCGGGGAGAGGCGGTTTGCGTATTGGGCGCTCTTCCGCTTCCTCGCTCA
CTGACTCGCTGCGCTCGGTCGTTCGGCTGCGGCGAGCGGTATCAGCTCACTCAAAGGCGGTAATACGGTTATCCACAGAA
TCAGGGGATAACGCNNAAAGAACATGTGAGCAAAAGGCCAGCAAAAGGCCAGGAACCGTAAAAAGGCCGCGTTGCTGGCG
TTTTTCCATNGGCTCCGCCCCCCTGACGAGCATCACAAAAATCGACGCTCAAGTCAGNNNGGNGAAACCCGACAGGACTA
TAAGATACCAGGCGTTTCCCCCNGGAAGCTCCCNCNNGCGCTCTCNNNTNCGACCCNNNNNTANNNNACNNTCNNNTNNN
CNNNCGGGANCNNNNNCTTTCTCNNANCNNNNNNCNNNANGNNTCTCANNNGNNNTNNNCNNNNNNNNNNNNNGNCNNNNN
NNCNNNNNNNCCNNNNNNNNCCNANNGNNNNTNNNNNCC

>mm9_dna range=chr9:71822294-71824207 5'pad=500 3'pad=500 strand=+
repeatMasking=none
TTATGTAAAGTAAATCCATCTGGATGTTTGCTCCATCCCATACTAGAAAC
CTGTGATCCCCAACCAGAATGAGTTATAGGAAAGCTGCAGGCAAAATTGT
CTCCATACAGCACAATCCATTATCCCTGTCAAGTTAATGTTCCGTGTTGA
GCCATATGGCACGGGTACACATTCAACAAAGGAAATTCCTTAAACTTGTT
ATAATCAGCACTTGCAGTCATCCAAAAAACTGACATAAACAGCTTTATTG
TGTTTAGCACATTTCAGTTAATATCCATGACAAAAACAGGAAACAGTACA
GGAAAAGGAGGAGGAATGTGGGGAGCAGTGTTATTTAAAAAAATCACCAC
ACACACACACACACACCCTCAAACTGTGCATTTTATAATTAGGCTTTTGT
CATTGTTGCTGTTGGCCTAAGCAAGTGATTTTCAATTACAGTAAGCACAC
CAACCTCTCCAGAGTGATCTACTGGGGCAGACTTAAGAGGGAAAAAAATT
AATGACAGCAATTTTCCAT**GGTATGAGCAAAACTAAACCTTGAAATTCTA**
GTGATTCAGATAAATCAGTTCTGGCAAAATAACAAAACTCTAAATCCTTA
TATTTGTTTTCTTTCTACAGTCATTTTATAATTGTTAAAATGCAACTGAG
CTTTATTAAGGAAAAAGAAACCAACGCAATTTAAGTAAAGTATCTTCATA
TGCATTTAAGAGCAGGTCACAGAAAATCAAGGGCTCCCACAAGCCTTTGG
TACTCGGCACATCAACTCTAGTCAAGGTATAAGTAGAGCAGAACTTCATT
TCAGCCCCCGCAGTGACAACAATAAAATGCTGAGAAAACACACTATCAAA
TACTGAACTCAGCATCAATTTCTGTTAATTACAGCCTTGTCAGCAACGTG
ATTATTCAAACAACTCATGCAAATGTTAATGAGGCCTTATTTGCATATTT
ATTTTTTCCTTTGTTGAAATGTCATTGATTATTACATTCTACTATGATGA
ATGTGGCTGATGATGTGCTCTGATATGTAATTAGTCATTAGGTAGAATGA
ATAGATCAATTATGAAAAAGGACTAAGATTAAGAAATATCAAACAAGACC
GCCCAACGTAACTATAGAATCTTTTTTTAAAAGGACCTAATAAATAATTT
CTTGACATTTAAACTGCAACATCAGTAGTTTTCGATAAATAAAGGACTAC
TTTTGTGAGGTTAAATGAAGGCTTAAAAATACATCAGTACTCTGATAATC
CAATTACGATCACAGAAACCATCTCTAGAAAAAAACCTGAAAGCCACCTA
GAATTTAATTGTCAGGAAAACAAGCAGCAAAGGAGAATTGAATCTGTTTT
AAGCTCACACAATTCAGAAATAAAGTGTATCTTAATCTAGTGTGAGGTGG
AGACAAAATCTGTTAAGAAAGAGGCATATAATATCCTAGAGAGAGGGGCA

AGAACCTGATGGAGAATTACTATTCATAAAACAGGGTTAGCCAAAAAAAA
AAAACCAAAAAAACAAAAAAACAAAAAAAAAACCAAAACCCCTTTACATG
GTGACTTGTGCTCAGAGGAAAGGATTATTTAGCTCCTGAAACAGTTTGCT
GTAACTATCCAGCCAGTACTTGCTATTCGCAGTTGAAAAAAAAAAAAAAA
AAACCACATTACTGCAGACCAGATGGGAACATTCCACATGACCAAACCAA
TCAATCACCTCGGTGGGGCGCAGGTGCAACACAGCCGTGTAGCAACACAC
CATTTCACAGTCTATCGGGCACAAACACATGGCCACCAATCAATGGCACC
CTGAGGACCTCGGAGGGGGCAGGCTCTCTGGAGTCTTACTGGTCCATGCC
TGATGACTTCATAATCACACATCATTTCATTCACTAGAGGATACAAACTA
GCATTCTAATAACC

Sequence hs623KO founder #3:

>MS_432601-504_1-2455-topo-_T7_B02.ab1
NNNNNNNNNNGCNGCTCGAGCGGCCGCCAGTGTGATGGATATCTGCAGAATTCGCCCTTGAGCAGTGTTATTTAAAAAAA
TCACCACACACACACACACACACCCTCAAACTGTGCATTTTATAATTAGGCTTTTGTCATTGTTGCTGTTGGCCTAAGCA
AGTGATTTTCAATTACAGTAAGCACACCAACCTCTCCAGAGTGATCTACTGGGGCAGACTTAAGAGGGAAAAAAATTAAT
GACAGCAATTTTCCATGGACAGAAACCATCTCTAGAAAAAAACCTGAAAGCCACCTAGAATTTAATTGTCAGGAAAACAA
GCAGCAAAGGAGAATTGAATCTGTTTTAAGCTCACACAATTCAGAAATAAAGTGTATCTTAATCTAGTGTGAGGTGGAGA
CAAAATCTGTTAAGAAAGAGGCATATAATATCCTAGAGAGAGGGGCAAGAACCTGATGAAGGGCGAATTCCAGCACACTG
GCGGCCGTTACTAGTGGATCCGAGCTCGGTACCAAGCTTGATGCATAGCTTGAGTATTCTATAGTGTCACCTAAATAGCT
TGGCGTAATCATGGTCATAGCTGTTTCCTGTGTGAAATTGTTATCCGCTCACAATTCCACACAACATACGAGCCGGAAGC
ATAAAGTGTAAAGCCTGGGGTGCCTAATGAGTGAGCTAACTCACATTAATTGCGTTGCGCTCACTGCCCGCTTTCCAGTC
GGGAAACCTGTCGTGCCAGCTGCATTAATGAATCGGCCAACGCGCGGGGAGAGGCGGTTTGCGTATTGGGCGCTCTTCCG
CTTCCTCGCTCACTGACTCGCTGCGCTCGGTCGTTCGGCTGCGGCGAGCGGTATCAGCTCACTCAAAGGCGGTAATACGG
TTATCCACAGAATCAGGGGATAACGCANGAAAGAACATGTGAGCAAAAGGCCAGCAAAAGGNCAGGAACCGTAAAAAGGC
CGCGTTGCTGGCGTTTTTCCATAGGCTCCGCCCCCCTGACGAGCATCACAAAAATCGACGCTCAAGTCANAGNNGNGAAA
CCCGACAGGACTATAAAGATACCAGGCGTTTCCCCCTNNNGCTCCCTCNNGNGCTCNNCNGNTCCGANCCNNCNNTNNNN
ANNNNGTCNNNTTNTCNNNCGGANCGNGNNGCTTNCTCNNAGNNNANGCNNNANGGNANCNCANNNNNNNNNAGNCNNNN
NNNNNNNNNNNNNNNNNNNNNNNNCNCNNNANNNNANNNCNNNNNCCCTTNN

>mm9_dna range=chr9:71822294-71824207 5'pad=500 3'pad=500 strand=+
repeatMasking=none
TTATGTAAAGTAAATCCATCTGGATGTTTGCTCCATCCCATACTAGAAAC
CTGTGATCCCCAACCAGAATGAGTTATAGGAAAGCTGCAGGCAAAATTGT
CTCCATACAGCACAATCCATTATCCCTGTCAAGTTAATGTTCCGTGTTGA
GCCATATGGCACGGGTACACATTCAACAAAGGAAATTCCTTAAACTTGTT
ATAATCAGCACTTGCAGTCATCCAAAAAACTGACATAAACAGCTTTATTG
TGTTTAGCACATTTCAGTTAATATCCATGACAAAAACAGGAAACAGTACA
GGAAAAGGAGGAGGAATGTGGGGAGCAGTGTTATTTAAAAAAATCACCAC
ACACACACACACACACCCTCAAACTGTGCATTTTATAATTAGGCTTTTGT
CATTGTTGCTGTTGGCCTAAGCAAGTGATTTTCAATTACAGTAAGCACAC
CAACCTCTCCAGAGTGATCTACTGGGGCAGACTTAAGAGGGAAAAAAATT
AATGACAGCAATTTTCCATGG**TATGAGCAAAACTAAACCTTGAAATTCTA**
GTGATTCAGATAAATCAGTTCTGGCAAAATAACAAAACTCTAAATCCTTA
TATTTGTTTTCTTTCTACAGTCATTTTATAATTGTTAAAATGCAACTGAG
CTTTATTAAGGAAAAAGAAACCAACGCAATTTAAGTAAAGTATCTTCATA
TGCATTTAAGAGCAGGTCACAGAAAATCAAGGGCTCCCACAAGCCTTTGG
TACTCGGCACATCAACTCTAGTCAAGGTATAAGTAGAGCAGAACTTCATT
TCAGCCCCCGCAGTGACAACAATAAAATGCTGAGAAAACACACTATCAAA
TACTGAACTCAGCATCAATTTCTGTTAATTACAGCCTTGTCAGCAACGTG
ATTATTCAAACAACTCATGCAAATGTTAATGAGGCCTTATTTGCATATTT
ATTTTTTCCTTTGTTGAAATGTCATTGATTATTACATTCTACTATGATGA
ATGTGGCTGATGATGTGCTCTGATATGTAATTAGTCATTAGGTAGAATGA
ATAGATCAATTATGAAAAAGGACTAAGATTAAGAAATATCAAACAAGACC
GCCCAACGTAACTATAGAATCTTTTTTTAAAAGGACCTAATAAATAATTT
CTTGACATTTAAACTGCAACATCAGTAGTTTTCGATAAATAAAGGACTAC
TTTTGTGAGGTTAAATGAAGGCTTAAAAATACATCAGTACTCTGATAATC
CAATTACGATCACAGAAACCATCTCTAGAAAAAAACCTGAAAGCCACCTA
GAATTTAATTGTCAGGAAAACAAGCAGCAAAGGAGAATTGAATCTGTTTT

AAGCTCACACAATTCAGAAATAAAGTGTATCTTAATCTAGTGTGAGGTGG
AGACAAAATCTGTTAAGAAAGAGGCATATAATATCCTAGAGAGAGGGGCA
AGAACCTGATGGAGAATTACTATTCATAAAACAGGGTTAGCCAAAAAAAA
AAAACCAAAAAAACAAAAAAACAAAAAAAAAAACCAAAACCCCTTTACATG
GTGACTTGTGCTCAGAGGAAAGGATTATTTAGCTCCTGAAACAGTTTGCT
GTAACTATCCAGCCAGTACTTGCTATTCGCAGTTGAAAAAAAAAAAAAAA
AAACCACATTACTGCAGACCAGATGGGAACATTCCACATGACCAAACCAA
TCAATCACCTCGGTGGGGCGCAGGTGCAACACAGCCGTGTAGCAACACAC
CATTTCACAGTCTATCGGGCACAAACACATGGCCACCAATCAATGGCACC
CTGAGGACCTCGGAGGGGGCAGGCTCTCTGGAGTCTTACTGGTCCATGCC
TGATGACTTCATAATCACACATCATTTCATTCACTAGAGGATACAAACTA
GCATTCTAATAACC

Sequence hs623KO founder #4 (F0 = 2458):

>MS_432601-505_1-2458-topo-_T7_C02.ab1
NNNNNNNNNNGCNTGCTCGAGCGGCCGCCAGTGTGATGGATATCTGCAGAATTCGCCCTTGAGCAGTGTTATTTAAAAAA
ATCACCACACACACACACACACACCCTCAAACTGTGCATTTTATAATTAGGCTTTTGTCATTGTTGCTGTTGGCCTAAGC
AAGTGATTTTCAATTACAGTAAGCACACCAACCTCTCCAGAGTGATCTACTGGGGCAGACTTAAGAGGGAAAAAAATTAA
TGACAGCAATTTTCCATGGACGATCACAGAAACCATCTCTAGAAAAAAACCTGAAAGCCACCTAGAATTTAATTGTCAGG
AAAACAAGCAGCAAAGGAGAATTGAATCTGTTTTAAGCTCACACAATTCAGAAATAAAGTGTATCTTAATCTAGTGTGAG
GTGGAGACAAAATCTGTTAAGAAAGAGGCATATAATATCCTAGAGAGAGGGGCAAGAACCTGATGAAGGGCGAATTCCAG
CACACTGGCGGCCGTTACTAGTGGATCCGAGCTCGGTACCAAGCTTGATGCATAGCTTGAGTATTCTATAGTGTCACCTA
AATAGCTTGGCGTAATCATGGTCATAGCTGTTTCCTGTGTGAAATTGTTATCCGCTCACAATTCCACACAACATACGAGC
CGGAAGCATAAAGTGTAAAGCCTGGGGTGCCTAATGAGTGAGCTAACTCACATTAATTGCGTTGCGCTCACTGCCCGCTT
TCCAGTCGGGAAACCTGTCGTGCCAGCTGCATTAATGAATCGGCCAACGCGCGGGGAGAGGCGGTTTGCGTATTGGGCGC
TCTTCCGCTTCCTCGCTCACTGACTCGCTGCGCTCGGTCGTTCGGCTGCGGCGAGCGGTATCAGCTCACTCAAAGGCGGT
AATACGGTTATCCACAGAATCAGGGGATAACGCNNGAAAGAACATGTGAGCAAAAGGNCAGCAAAAGGCCAGGAACCGTA
AAAAGGCCGCGTTGCTGGCGTTTTTCCATAGGCTCCGCCCCCCTGACGAGCATCACAAAAATCGACGCTCAAGTCANAGG
TGGNGAAACCCCGACAGGACTATAAAGATACCAGGCGTTTCCCCNNNNNCTCCCTCNNGCNNNTCNNCNGTCNACCNNCN
CTTACNNATACNGTCNNNTNCTCCNTNGNNNCGNGGCGCTTNNNCATAGCTCANGCNNNAGNATCTCANNTCNNNNNNNG
NCNNNNNCTNNNNNNNNNNNNNNNNNNNCNANCNCNNNCNNTNCNNNCNCNNANCNNNNNNNNNNC

>mm9_dna range=chr9:71822294-71824207 5'pad=500 3'pad=500 strand=+
repeatMasking=none
TTATGTAAAGTAAATCCATCTGGATGTTTGCTCCATCCCATACTAGAAAC
CTGTGATCCCCAACCAGAATGAGTTATAGGAAAGCTGCAGGCAAAATTGT
CTCCATACAGCACAATCCATTATCCCTGTCAAGTTAATGTTCCGTGTTGA
GCCATATGGCACGGGTACACATTCAACAAAGGAAATTCCTTAAACTTGTT
ATAATCAGCACTTGCAGTCATCCAAAAAACTGACATAAACAGCTTTATTG
TGTTTAGCACATTTCAGTTAATATCCATGACAAAAACAGGAAACAGTACA
GGAAAAGGAGGAGGAATGTGGGGAGCAGTGTTATTTAAAAAAATCACCAC
ACACACACACACACACCCTCAAACTGTGCATTTTATAATTAGGCTTTTGT
CATTGTTGCTGTTGGCCTAAGCAAGTGATTTTCAATTACAGTAAGCACAC
CAACCTCTCCAGAGTGATCTACTGGGGCAGACTTAAGAGGGAAAAAAATT
AATGACAGCAATTTTCCATGG**TATGAGCAAAACTAAACCTTGAAATTCTA**
GTGATTCAGATAAATCAGTTCTGGCAAAATAACAAAACTCTAAATCCTTA
TATTTGTTTTCTTTCTACAGTCATTTTATAATTGTTAAAATGCAACTGAG
CTTTATTAAGGAAAAAGAAACCAACGCAATTTAAGTAAAGTATCTTCATA
TGCATTTAAGAGCAGGTCACAGAAAATCAAGGGCTCCCACAAGCCTTTGG
TACTCGGCACATCAACTCTAGTCAAGGTATAAGTAGAGCAGAACTTCATT
TCAGCCCCCGCAGTGACAACAATAAAATGCTGAGAAAACACACTATCAAA
TACTGAACTCAGCATCAATTTCTGTTAATTACAGCCTTGTCAGCAACGTG
ATTATTCAAACAACTCATGCAAATGTTAATGAGGCCTTATTTGCATATTT
ATTTTTTCCTTTGTTGAAATGTCATTGATTATTACATTCTACTATGATGA
ATGTGGCTGATGATGTGCTCTGATATGTAATTAGTCATTAGGTAGAATGA
ATAGATCAATTATGAAAAAGGACTAAGATTAAGAAATATCAAACAAGACC
GCCCAACGTAACTATAGAATCTTTTTTTAAAAGGACCTAATAAATAATTT
CTTGACATTTAAACTGCAACATCAGTAGTTTTCGATAAATAAAGGACTAC
TTTTGTGAGGTTAAATGAAGGCTTAAAAATACATCAGTACTCTGATAATC
CAATTACGATCACAGAAACCATCTCTAGAAAAAAACCTGAAAGCCACCTA

GAATTTAATTGTCAGGAAAACAAGCAGCAAAGGAGAATTGAATCTGTTTT
AAGCTCACACAATTCAGAAATAAAGTGTATCTTAATCTAGTGTGAGGTGG
AGACAAAATCTGTTAAGAAAGAGGCATATAATATCCTAGAGAGAGGGGCA
AGAACCTGATGGAGAATTACTATTCATAAAACAGGGTTAGCCAAAAAAAA
AAAACCAAAAAAACAAAAAAACAAAAAAAAAACCAAAACCCCTTTACATG
GTGACTTGTGCTCAGAGGAAAGGATTATTTAGCTCCTGAAACAGTTTGCT
GTAACTATCCAGCCAGTACTTGCTATTCGCAGTTGAAAAAAAAAAAAAA
AAACCACATTACTGCAGACCAGATGGGAACATTCCACATGACCAAACCAA
TCAATCACCTCGGTGGGGCGCAGGTGCAACACAGCCGTGTAGCAACACAC
CATTTCACAGTCTATCGGGCACAAACACATGGCCACCAATCAATGGCACC
CTGAGGACCTCGGAGGGGGCAGGCTCTCTGGAGTCTTACTGGTCCATGCC
TGATGACTTCATAATCACACATCATTTCATTCACTAGAGGATACAAACTA
GCATTCTAATAACC

Sequence hs623KO founder #5:

>MS_432601-506_1-2459-topo-_T7_D02.ab1
NNNNNNNNNNGNNNGCTCGAGCGGCCGCCAGTGTGATGGATATCTGCAGAATTCGCCCTTGAGCAGTGTTATTTAAAAAA
ATCACCACACACACACACACACACCCTCAAACTGTGCATTTTATAATTAGGCTTTTGTCATTGTTGCTGTTGGCCTAAGC
AAGTGATTTTCAATTACAGTAAGCACACCAACCTCTCCAGAGTGATCTACTGGGGCAGACTTAAGAGGGAAAAAAATTAA
TGACAGCAATTTTCTGTGATCACAGAAACCATCTCTAGAAAAAAACCTGAAAGCCACCTAGAATTTAATTGTCAGGAAAA
CAAGCAGCAAAGGAGAATTGAATCTGTTTTAAGCTCACACAATTCAGAAATAAAGTGTATCTTAATCTAGTGTGAGGTGG
AGACAAAATCTGTTAAGAAAGAGGCATATAATATCCTAGAGAGAGGGGCAAGAACCTGATGAAGGGCGAATTCCAGCACA
CTGGCGGCCGTTACTAGTGGATCCGAGCTCGGTACCAAGCTTGATGCATAGCTTGAGTATTCTATAGTGTCACCTAAATA
GCTTGGCGTAATCATGGTCATAGCTGTTTCCTGTGTGAAATTGTTATCCGCTCACAATTCCACACAACATACGAGCCGGA
AGCATAAAGTGTAAAGCCTGGGGTGCCTAATGAGTGAGCTAACTCACATTAATTGCGTTGCGCTCACTGCCCGCTTTCCA
GTCGGGAAACCTGTCGTGCCAGCTGCATTAATGAATCGGCCAACGCGCGGGGAGAGGCGGTTTGCGTATTGGGCGCTCTT
CCGCTTCCTCGCTCACTGACTCGCTGCGCTCGGTCGTTCGGCTGCGGCGAGCGGTATCAGCTCACTCAAAGGCGGTAATA
CGGTTATCCACAGAATCANGGGATAACGCAGGAAAGAACATGTGAGCAAAAGGCCAGCAAAAGGCCAGGAACCGTAAAAA
GGCCGCGTTGCTGGCGTTTTTCCATAGGCTCCGCCCCCCTGACGAGCATCACAAAAATCGACGCTCAAGTCANANNNGGC
GAAACCCGACAGGACTATAAAGANACCAGGCGTTTCCCCNGNNNNNTCCCNNNNNNNNNTCNNNNNNNNNNANCCNNCNCT
ACNGNATACNGTCNCNTTCTCCNNGGNANNNNNNNGGNGCTTNCNCATANCTNNNGCNNNANGNNNNNNANNNNNGNNNAN
GNCNNNNNNNNNNNNNCNGGNNNNGNNNNNNNNNNCNNNNNNCANNCNNANNNNNNNNNNNNN

>mm9_dna range=chr9:71822294-71824207 5'pad=500 3'pad=500 strand=+ repeatMasking=none
TTATGTAAAGTAAATCCATCTGGATGTTTGCTCCATCCCATACTAGAAAC
CTGTGATCCCCAACCAGAATGAGTTATAGGAAAGCTGCAGGCAAAATTGT
CTCCATACAGCACAATCCATTATCCCTGTCAAGTTAATGTTCCGTGTTGA
GCCATATGGCACGGGTACACATTCAACAAAGGAAATTCCTTAAACTTGTT
ATAATCAGCACTTGCAGTCATCCAAAAAACTGACATAAACAGCTTTATTG
TGTTTAGCACATTTCAGTTAATATCCATGACAAAAACAGGAAACAGTACA
GGAAAAGGAGGAGGAATGTGGGGAGCAGTGTTATTTAAAAAAATCACCAC
ACACACACACACACACCCTCAAACTGTGCATTTTATAATTAGGCTTTTGT
CATTGTTGCTGTTGGCCTAAGCAAGTGATTTTCAATTACAGTAAGCACAC
CAACCTCTCCAGAGTGATCTACTGGGGCAGACTTAAGAGGGAAAAAAATT
AATGACAGCAATTTTC**CATGGTATGAGCAAAACTAAACCTTGAAATTCTA**
GTGATTCAGATAAATCAGTTCTGGCAAAATAACAAAACTCTAAATCCTTA
TATTTGTTTTCTTTCTACAGTCATTTTATAATTGTTAAAATGCAACTGAG
CTTTATTAAGGAAAAAGAAACCAACGCAATTTAAGTAAAGTATCTTCATA
TGCATTTAAGAGCAGGTCACAGAAAATCAAGGGCTCCCACAAGCCTTTGG
TACTCGGCACATCAACTCTAGTCAAGGTATAAGTAGAGCAGAACTTCATT
TCAGCCCCCGCAGTGACAACAATAAAATGCTGAGAAAACACACTATCAAA
TACTGAACTCAGCATCAATTTCTGTTAATTACAGCCTTGTCAGCAACGTG
ATTATTCAAACAACTCATGCAAATGTTAATGAGGCCTTATTTGCATATTT
ATTTTTTCCTTTGTTGAAATGTCATTGATTATTACATTCTACTATGATGA
ATGTGGCTGATGATGTGCTCTGATATGTAATTAGTCATTAGGTAGAATGA
ATAGATCAATTATGAAAAAGGACTAAGATTAAGAAATATCAAACAAGACC
GCCCAACGTAACTATAGAATCTTTTTTTAAAAGGACCTAATAAATAATTT
CTTGACATTTAAACTGCAACATCAGTAGTTTCGATAAATAAAGGACTAC

TTTTGTGAGGTTAAATGAAGGCTTAAAAATACATCAGTACTCTGATAATC
CAATTACGATCACAGAAACCATCTCTAGAAAAAAACCTGAAAGCCACCTA
GAATTTAATTGTCAGGAAAACAAGCAGCAAAGGAGAATTGAATCTGTTTT
AAGCTCACACAATTCAGAAATAAAGTGTATCTTAATCTAGTGTGAGGTGG
AGACAAAATCTGTTAAGAAAGAGGCATATAATATCCTAGAGAGAGGGGCA
AGAACCTGATGGAGAATTACTATTCATAAAACAGGGTTAGCCAAAAAAAA
AAAACCAAAAAAACAAAAAAACAAAAAAAAAAACCAAAACCCCTTTACATG
GTGACTTGTGCTCAGAGGAAAGGATTATTTAGCTCCTGAAACAGTTTGCT
GTAACTATCCAGCCAGTACTTGCTATTCGCAGTTGAAAAAAAAAAAAAAA
AAACCACATTACTGCAGACCAGATGGGAACATTCCACATGACCAAACCAA
TCAATCACCTCGGTGGGGCGCAGGTGCAACACAGCCGTGTAGCAACACAC
CATTTCACAGTCTATCGGGCACAAACACATGGCCACCAATCAATGGCACC
CTGAGGACCTCGGAGGGGGCAGGCTCTCTGGAGTCTTACTGGTCCATGCC
TGATGACTTCATAATCACACATCATTTCATTCACTAGAGGATACAAACTA
GCATTCTAATAACC
